# Development of 17 microsatellite markers in the federally endangered species *Liatris helleri* (Asteraceae)

**DOI:** 10.1002/aps3.1250

**Published:** 2019-05-17

**Authors:** Logan C. Clark, Morgan R. Gaglianese‐Woody, Matt C. Estep

**Affiliations:** ^1^ Department of Biology Appalachian State University 572 Rivers Street Boone North Carolina 28607‐2027 USA

**Keywords:** Asteraceae, endangered species, *Liatris helleri*, perennial herb, Southern Appalachians, species boundaries

## Abstract

**Premise:**

Microsatellite markers were developed in the federally endangered species *Liatris helleri* (Asteraceae) to evaluate species boundaries with closely related congeners within the genus.

**Methods and Results:**

Using Illumina data, 17 primer pairs were developed in populations of *L. helleri*. The primers amplified motifs from tri‐ to hexanucleotide repeats with one to 17 alleles per locus. Primers were also tested for cross‐amplification in *L. aspera*,* L. microcephala*, and *L. pycnostachya*.

**Conclusions:**

The developed primers for *L. helleri* serve as a novel genetic tool for future investigations in this genus, allowing for more explicit species delineation as well as population genetic analyses.

The North American genus *Liatris* Gaertn. ex Schreb. (Asteraceae, Asterales) is composed of 40–50 species, mainly confined to the eastern seaboard of North America (Gaiser, 1946; Weakley, 2015). *Liatris* has been considered a genus of “unusual difficulty” due to variability and hybridization between species that has led to unclear delineation of species boundaries (Gaiser, 1946). Species of *Liatris* are broadly sympatric, but ecologically distinct in their distribution, which is related to gradients of available nutrients, soil moistures, and elevation (Levin, 1967). Phenology in the genus occurs mostly in late summer through early fall, but periods of overlap in seasonal phenology even between the earliest and latest flowering species may facilitate hybridization in areas of sympatry (Levin, 1967). Morphological distinctions in this genus are not abundant and have led to somewhat blurry species delineation. This has been the case with *L. helleri* Porter and its closely related congener *L. turgida* Gaiser (Gaiser, 1946; Nesom, 2005a). Because *L. helleri* is listed as federally endangered, it is crucial for land managers and conservationists alike to have a clear concept of the boundaries of this species.

## METHODS AND RESULTS

DNA was extracted from a single individual of *L. helleri* (BOON28026; Appendix 1) using a modified cetyltrimethylammonium bromide (CTAB) method (Doyle and Doyle, 1987). An Illumina MiSeq sequencing library was constructed and paired‐end sequenced at the West Virginia University Genomics Core Facility. Raw sequence reads were quality controlled and trimmed using fastp (Chen et al., 2018). A total of 18,020,464 sequence reads were queried by MSATCOMMANDER version 1.0.8 (Faircloth, 2008) with default settings, minimum primer size was set at 20 bp, maximum primer GC content was limited to 50%, and a PIG‐tail sequence (GTTT) (Brownstein et al., 1996) was added to one primer. A total of 192,645 microsatellite loci were identified, 6919 of which were suitable for primer design.

Three populations, each composed of multiple subpopulations, were sampled by collecting single leaf samples from individuals (Appendix 1). Samples were then stored on silica gel and placed in a −80°C freezer until used for DNA extraction. Extractions were performed using the PureLink Plant Total DNA Purification Kit (Invitrogen, Carlsbad, California, USA). One hundred and nineteen primer pairs were tested by amplifying under standard conditions in a group of seven individuals and a negative control. PCR reactions were prepared in 10‐μL volumes consisting of 1× Go Taq Flexi Buffer, 2.5 mM MgCl_2_, 800 μM dNTPs, 0.5 μM each primer, 0.5 units Go Taq Flexi DNA Polymerase (Promega Corporation, Madison, Wisconsin, USA), and 1 μL of DNA. PCR was completed using a touchdown thermal cycling program on an Eppendorf Mastercycler (Eppendorf, Hauppauge, New York, USA) with annealing temperatures ranging from 68°C to 55°C. Initial denaturation was 94°C for 5 min, followed by 13 cycles (45 s at 94°C, 2 min at annealing temperature, and 1 min at 72°C), followed by 24 cycles (45 s at 94°C, 1 min at 55°C, and 1 min at 72°C), followed by 10 min at 72°C. PCR products were examined on a 1% agarose gel and scored for the presence or absence of an appropriately sized PCR product and uniform amplification across samples. A total of 20 primers consistently amplified and were further examined by pseudo‐multiplexing fluorescently labeled PCR products with 6‐FAM, VIC, NED, or PET by adding 0.25 μM of an M13 primer (5′‐CACGACGTTGTAAAACGAC‐3′) to the PCR reaction following Schuelke (2000). PCR products were pooled and combined with a GeneScan 500 LIZ Size Standard (Life Technologies, Carlsbad, California, USA) for genotyping on an ABI 3730xl DNA Analyzer at the Georgia Genomics Facility (Athens, Georgia, USA). Resulting chromatograms were scored using Geneious 9.1.5 (Kearse et al., 2012; Biomatters Ltd., Auckland, New Zealand). Markers displaying more than two alleles for a single individual or failing to be easily scorable were removed from further analysis. The resulting genotypic data were analyzed using GenAlEx version 6.503 (Peakall and Smouse, 2012) to obtain standard descriptive statistics and test for per population deviations of Hardy–Weinberg equilibrium at each locus. The presence of null alleles was tested using MICRO‐CHECKER (van Oosterhout et al., 2004). Tests for linkage disequilibrium and global exact tests of heterozygosity deficiency were performed in GENEPOP using default Markov chain parameters (Rousset, 2008).

Seventeen of the primer pairs consistently amplified and produced chromatograms that were easily scored. Three of these markers (LH2, LH4, and LH24) were monomorphic (Table 1). The remaining 14 polymorphic markers produced from two to 17 alleles per locus with an average of 6.0 (Table 2). The effective number of alleles per locus ranged from 1.09 to 10.00 with an average of 3.38 (Table 2). Expected levels of heterozygosites ranged from 0.083–0.900 with an average of 0.640 (Table 2). Markers LH14, LH21, LH22, LH68, and LH78 showed evidence for the presence of null alleles. Observed levels of heterozygosities tended to be lower than expected, which aligns with results from a previous study using allozyme markers (Godt and Hamrick, 1996). The excess of homozygotes indicated by a global exact test (*P* < 0.000) was not consistent across populations and could also be due to the Wahlund effect caused by sampling very small subpopulations of this federally listed species (Table 2). Significant linkage disequilibrium was detected between marker pair LH22/LH83 (*P* < 0.001) and marker pairs LH10/LH21, LH10/LH22, LH25/LH67, and LH16/LH69 (*P* < 0.05).

**Table 1:** Characteristics of 17 microsatellite markers developed for *Liatris helleri*

Locus	Primer sequences (5′–3′)	Repeat motif	Allele size range (bp)	*T*_a_ (°C)	Fluorescent label	GenBank accession no.
LH2	F: ACACCAACAATGACATCCTGC	(AAAAG)_6_	187 M	59	NED	MK246216
	R: GTTTGAAGTACAGACCCAATACACC					
LH4	F: GGGAAATTGTGCGCTTAGTTTG	(AAAAT)_6_	133 M	59	VIC	MK246217
	R: GTTTCACACTTAACACACCTTGCG					
LH10	F: GTTTCTTGCGAGGCCTTCTTTC	(AAAG)_6_	126–146	59	FAM	MK246218
	R: TCGGGTTCAAATCATGGAATCC					
LH14	F: TTTCGGTAAGCAGGTTCCCATC	(AAATAT)_6_	210–234	60	VIC	MK246219
	R: GTTTCTCTCCACTTTCCCAGAAAC					
LH16	F: GATGCCAACACAGGTAAACATC	(AAATGT)_7_	225–243	59	NED	MK246220
	R: GTTTATACCGGCATAACTTTCGCC					
LH21	F: GTTTGTATCATCACACACAGTCGG	(AACAAT)_9_	258–295	59	FAM	MK246221
	R: AGCCTGCCTATGATTGTACTCC					
LH22	F: ATGCCTCGTTGTTGATGGTC	(AACAAT)_6_	203–305	59	VIC	MK246222
	R: GTTTCAAAGTGGGACTGGTAGC					
LH24	F: TGTGCTTGTTCCTGTTCCAG	(AACAGG)_6_	137 M	59	FAM	MK246223
	R: GTTTAAACCGCATACTGTGAAAGATG					
LH25	F: GTTTAACCGTTTCTCCTAATCCGC	(AACC)_6_	218–238	59	FAM	MK246224
	R: TGGAGACGAGTACCAGAACTAC					
LH67	F: TCCTATGTGATCCCTGTGTGTC	(ATC)_15_	192–236	59	VIC	MK246225
	R: GTTTAAGGCTGTCTACGTCTTACCC					
LH68	F: AGGTTATCACGGTTTAGCGC	(ATGC)_6_	121–133	60	PET	MK246226
	R: GTTTCCGGTCAGCATGTCTAC					
LH69	F: ATCTGGTGAAGGTGTGACTACC	(CCG)_8_	181–208	59	PET	MK246227
	R: GTTTCAGAGGCAGAAGGTTTGG					
LH78	F: GTTTGTGCTTGCTCCCTAACAAC	(AAC)_9_	185–244	59	NED	MK246228
	R: ATGACGTGATTGCTGCTGTG					
LH82	F: AAGCGCAAAGATTGTCCCAC	(AAG)_12_	259–334	60	VIC	MK246229
	R: GTTTCATCAATCGGTTTCACGCC					
LH83	F: TGATCAAGGCCGGCATATTG	(AAG)_10_	136–168	59	PET	MK246230
	R: GTTTAGAGAGTTGGATCAAGGACATG					
LH84	F: AAAGCATTGCGAGAAGAGGG	(AAG)_11_	103–125	59	PET	MK246231
	R: GTTTAATAGCGCGCTGAAGAGTG					
LH89	F: GTTTCTTCTTCATCATGTCGCCTG	(AATAT)_7_	137–211	59	PET	MK246232
	R: GGACAAATAACCGATCCGATCC					

Note

M = monomorphic; *T*_a_ = annealing temperature.

**Table 2:** Descriptive statistics for 14 polymorphic microsatellite markers in three populations of *Liatris helleri*.^a^

Locus	Blue Ridge Parkway (*n* = 36)				Linville (*n* = 30)				Shortoff (*n* = 20)			
	*A*	*A*_e_	*H*_o_	*H*_e_	*A*	*A*_e_	*H*_o_	*H*_e_	*A*	*A*_e_	*H*_o_	*H*_e_
LH10	4	2.65	0.133*	0.622	3	1.16	0.143	0.135	4	3.60	0.133*	0.722
LH14	6	3.95	0.571	0.747	6	3.64	0.619*	0.726	5	2.75	0.579	0.636
LH16	5	3.07	0.567*	0.674	4	1.48	0.385	0.323	4	2.23	0.389	0.551
LH21	5	3.15	0.190*	0.683	3	2.13	0.000*	0.531	5	3.03	0.083*	0.670
LH22	2	1.09	0.000*	0.083	6	3.35	0.200*	0.701	3	2.77	0.308*	0.639
LH25	6	2.50	0.458*	0.601	5	3.81	0.792	0.738	5	3.93	0.500*	0.745
LH67	8	3.98	0.500	0.749	11	6.90	0.346*	0.855	4	3.06	0.200*	0.673
LH68	5	3.09	0.440*	0.676	5	4.02	0.133*	0.751	3	1.92	0.000*	0.480
LH69	7	3.17	0.379*	0.685	4	2.08	0.115*	0.518	4	2.34	0.158*	0.572
LH78	8	3.83	0.423*	0.739	5	4.25	0.318*	0.764	6	5.02	0.688*	0.801
LH82	17	10.00	0.667*	0.900	15	8.01	0.600*	0.875	9	4.78	0.789	0.791
LH83	3	2.07	0.833*	0.517	5	2.04	0.680	0.510	3	2.10	1.000*	0.525
LH84	6	2.87	0.464*	0.651	6	4.17	0.760	0.760	5	3.38	0.350*	0.704
LH89	5	2.26	0.280	0.557	5	2.10	0.250*	0.524	7	4.35	0.350*	0.770

Note

*A* = number of alleles; *A*_e_ = effective number of alleles; *H*_e_ = expected heterozygosity; *H*_o_ = observed heterozygosity; *n* = number of individuals sampled.

a Population and voucher information are provided in Appendix 1.

* Significant deviation from Hardy–Weinberg equilibrium (*P* < 0.05).

Cross‐amplification experiments were performed by extracting DNA from five individuals from each of three species: *L. aspera* Michx., *L. microcephala* (Small) K. Schum., and *L. pycnostachya* Michx. (Table 3, Appendix 1). Each species was chosen to represent a different clade within the genus (Nesom, 2005b). Most of the markers cross‐amplified in all three species, but markers LH10 and LH68 failed to cross‐amplify and markers LH21, LH24, LH67, and LH84 were not 100% effective.

**Table 3:** Cross‐amplification of 17 primer pairs developed in *Liatris helleri* in three other *Liatris* species.^a^^,^ b

Locus	*L. aspera*	*L. pycnostachya*	*L. microcephala*
LH2	100%	100%	100%
LH4	100%	100%	100%
LH10	—	—	—
LH14	100%	100%	100%
LH16	100%	100%	100%
LH21	60%	20%	—
LH22	100%	100%	100%
LH24	60%	60%	—
LH25	100%	100%	100%
LH67	60%	—	100%
LH68	—	—	—
LH69	100%	100%	100%
LH78	100%	100%	100%
LH82	100%	100%	100%
LH83	100%	100%	100%
LH84	100%	60%	100%
LH89	100%	100%	100%

Note

— = unsuccessful amplification.

a Population and voucher information are provided in Appendix 1.

b Percentage of five individuals that successfully amplified an appropriately sized product for the locus.

## CONCLUSIONS

The 17 microsatellite markers developed here will be a useful tool to investigate the genetic diversity of *L. helleri* species and can be used to better understand species boundaries between *L. helleri* and *L. turgida*. These markers also displayed the ability to cross‐amplify in *L. aspera*,* L. microcephala*, and *L. pycnostachya*, each representing distinct clades within the genus, suggesting these markers will provide the ability to assess genetic diversity of these species. The application of these markers should lead to a more thorough understanding of the dynamic properties of this genus while providing data for more efficient management and conservation strategies.

## Data Availability

Sequence information for the developed primers has been deposited to the National Center for Biotechnology Information (NCBI); GenBank accession numbers are provided in Table 1.

## APPENDIX 1 Voucher information for the specimens used in this study. All specimens are deposited in the I. W. Carpenter Jr. Herbarium at Appalachian State University (BOON).^a^

**Table:**

Species	Population	No. of samples represented	Herbarium accession no.	Collector
*L. helleri* Porter	Shortoff	20	BOON28016	P. Sullins & G. Kauffman
*L. helleri*	Linville	30	BOON28017	P. Sullins
*L. helleri*	Blue Ridge Parkway	36	BOON28026	P. Sullins
*L. aspera* Michx.	Gardens of the Blue Ridge	5	BOON30483	L. Clark
*L. microcephala* (Small) K. Schum.	Gardens of the Blue Ridge	5	BOON30484	L. Clark
*L. pycnostachya* Michx.	Gardens of the Blue Ridge	5	BOON30485	L. Clark

a GPS coordinates are not provided in the interest of protecting locality information for this federally listed species.
